# Supplementary material for: Elevated levels of Bcl-3 inhibits Treg development and function resulting in spontaneous colitis
Source: Nat Commun. 2017 Apr 28;8:15069. doi: 10.1038/ncomms15069 (PMC5414353; doi:10.1038/ncomms15069)
Supplement: Supplementary Information — Supplementary Figures [file ncomms15069-s1.pdf]

Supplementary Figure 1

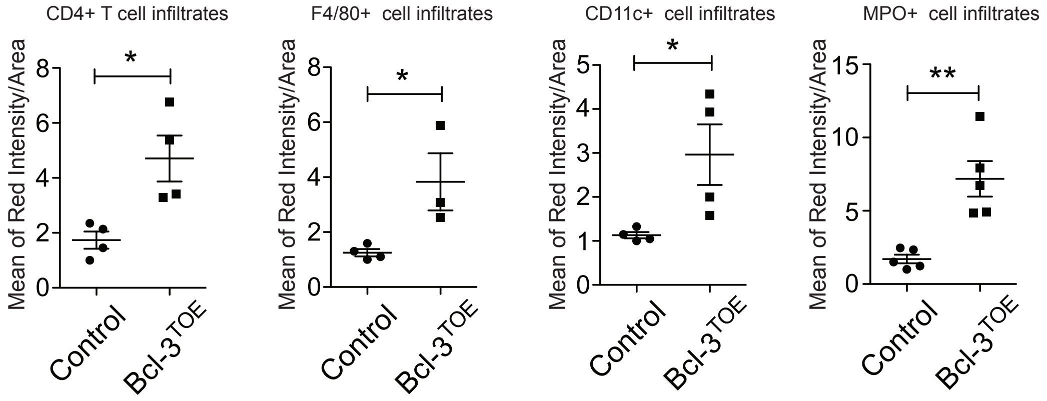

Supplementary Figure 1: Increased immune cell infiltration in the colon of 8 weeks old colitic Bcl-3^{TOE} mice
The graph display mean of red intensity/area of colonic cryoslides stained for CD4^{+} T cells, F4/80^{+}, CD11c^{+} and MPO^{+} cell infiltrates. Colons were isolated from 8 weeks old colitic Bcl-3^{TOE} mice and 8 weeks old littermate controls (n = 4 - 5). Quantification was performed using ImageJ. Mean ± SEM. *p < 0.05, **p < 0.01 using unpaired Student´s t-test. Bcl-3^{OE} mice without Cre were used as littermate controls.

Supplementary Figure 2

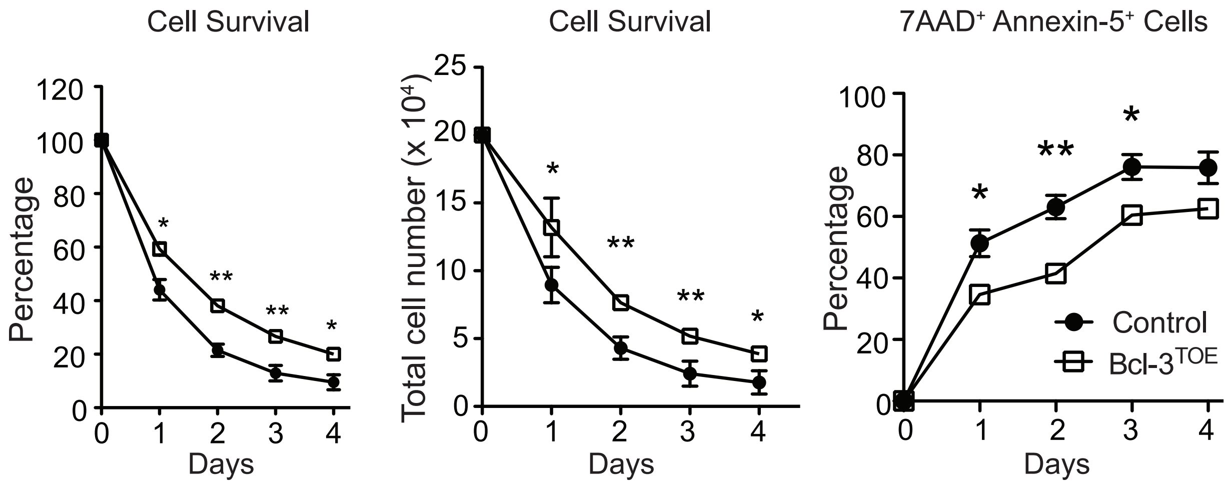

Supplementary Figure 2: Bcl-3 promotes T cell survival

MACS purified $CD4^+$ T cells were isolated from spleen (Spl) and lymph node (LN) of Bcl-3^{TOE} mice (n = 3) and littermate controls (n = 3). $CD4^+$ T cells were left untreated in culture at 37 °C, counted and stained for AnnexinV and 7AAD at the indicated days (1, 2, 3 and 4) and analyzed by flow cytometry. Graphs represent the percentage of $7AAD^-$ $AnnnexinV^-$ living cells (left graph), the total cell number of $7AAD^-$ $AnnnexinV^-$ living cells (middle graph) and the percentage of $7AAD^+$ $AnnnexinV^+$ apoptotic cells (right graph). Mean ± SEM. *$p < 0.05$, **$p < 0.01$ using unpaired Student's t-test. Data shown are representative of two independent experiments (n = 3).

Supplementary Figure 3

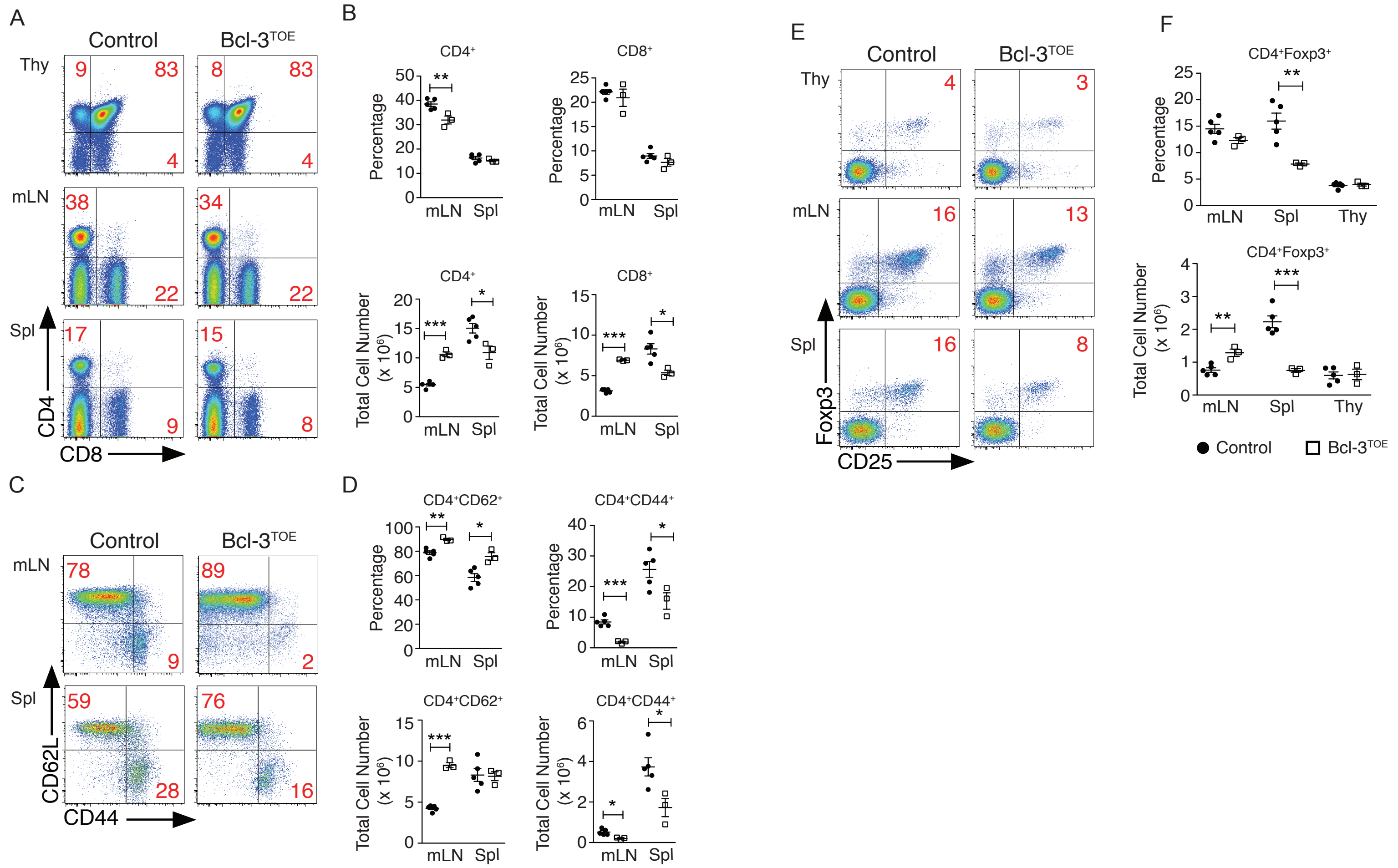

Supplementary Figure 3: T cell homeostasis in peripheral lymphoid organs of four weeks old Bcl-3^{TOE} mice
(a) Representative flow cytometric analysis of CD4^{+} and CD8^{+} T cells in thymus (Thy), mesenteric lymph nodes (mLN) and spleen (Spl) of Bcl-3^{TOE} (n = 3) and littermate controls (n = 5). (b) Percentage (upper panel) and absolute cell numbers (lower panel) of CD4^{+} and CD8^{+} T cells, respectively. (c) Representative flow cytometric analysis of naïve and effector CD4^{+} T cells as determined by CD62L and CD44 expression in mLN and Spl from Bcl-3^{TOE} (n = 3) and littermate controls (n = 5). (d) Percentage (upper panel) and total cell numbers (lower panel) of naïve and effector CD4^{+} T cells as determined by CD62L and CD44 expression, respectively. (e) Representative flow cytometric analysis of regulatory T cells in Thy, mLN and Spl from Bcl- 3^{TOE} (n = 3) and littermate controls (n = 5). (f) Percentage (upper panel) and absolute cell numbers (lower panel) of CD4^{+} Foxp3^{+} Tregs of Bcl-3^{TOE} mice compared to littermate controls. Numbers in quadrants indicate percentage. Data shown are representative of at least three independent experiments. Each symbol represents one single mouse. *$p < 0.05$, **$p < 0.01$, ***$p < 0.001$ using unpaired Student ́s t-test. Bcl-3^{OE} mice without Cre were used as control littermates.

Supplementary Figure 4

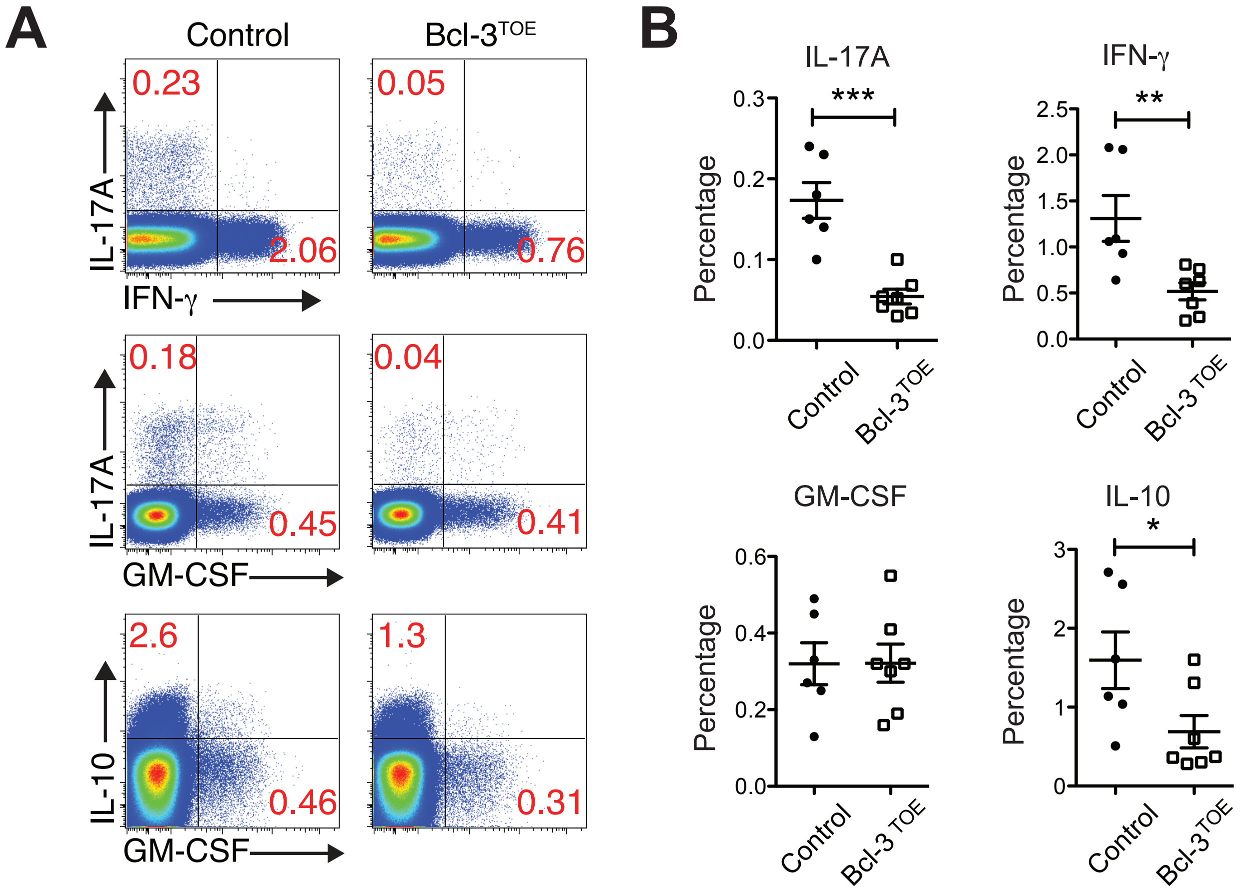

Supplementary Figure 4: Decreased cytokine expression by T cells isolated from Bcl-3^{TOE} mice

(a) Representative flow cytometric analysis of cytokine expression by CD4^{+} T cells isolated from Spl and LN of the indicated mice (n = 3). Cells are gated on CD4. Data are shown as representative FACS plots. Numbers in the quadrants represent percentage. Data shown are representative of two independent experiments (n = 3 - 4). (b) Percentage of cytokine expression by CD4^{+} T cells from mice with the indicated genotypes. Each symbol represents one single mouse. Data are pooled from two independent experiments (n = 3 - 4). Mean ± SEM. *$p < 0.05$, **$p < 0.01$, ***$p < 0.001$ using unpaired Student's t-test. Bcl-3^{OE} mice without Cre were used as littermate controls.

Supplementary Figure 5

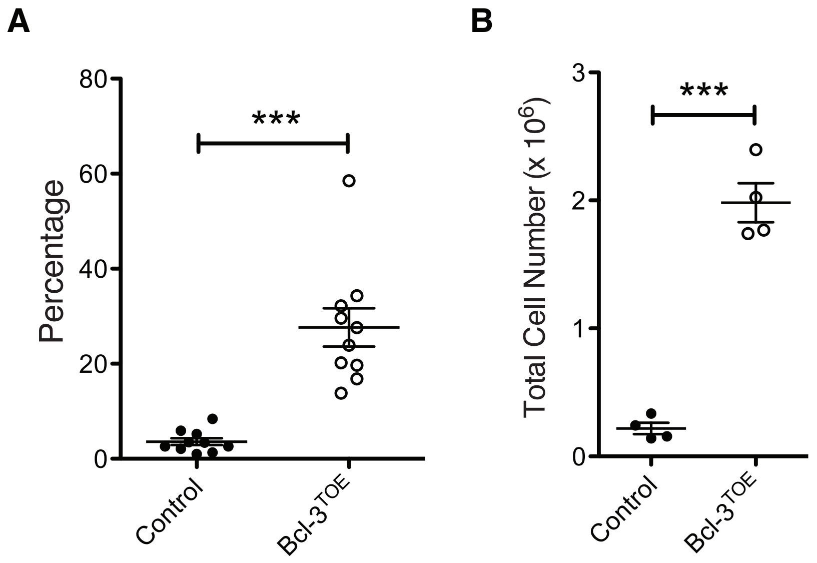

Supplementary Figure 5: Increased numbers of γδ T cells in the colonic IEL compartment of Bcl-3^{TOE} mice

(a) Graph displays mean percentages of CD8α^{+} γδ T cells in the IEL compartment from the colon of Bcl-3^{TOE} mice and littermate controls. Mean ± SEM. ***$p < 0.001$ using unpaired Student's t-test, n = 10. Data are pooled from three independent experiments (n = 3 - 4). (b) Mean total cell numbers of CD8α^{+} γδ T cells in the IEL compartment from the colon of Bcl-3^{TOE} mice and littermate controls. Mean ± SEM. ***$p < 0.001$ using unpaired Student's t-test. N = 4. Data shown are representative of three independent experiments (n = 3 - 4). Bcl-3^{OE} mice without Cre were used as littermate controls.

Supplementary Figure 6

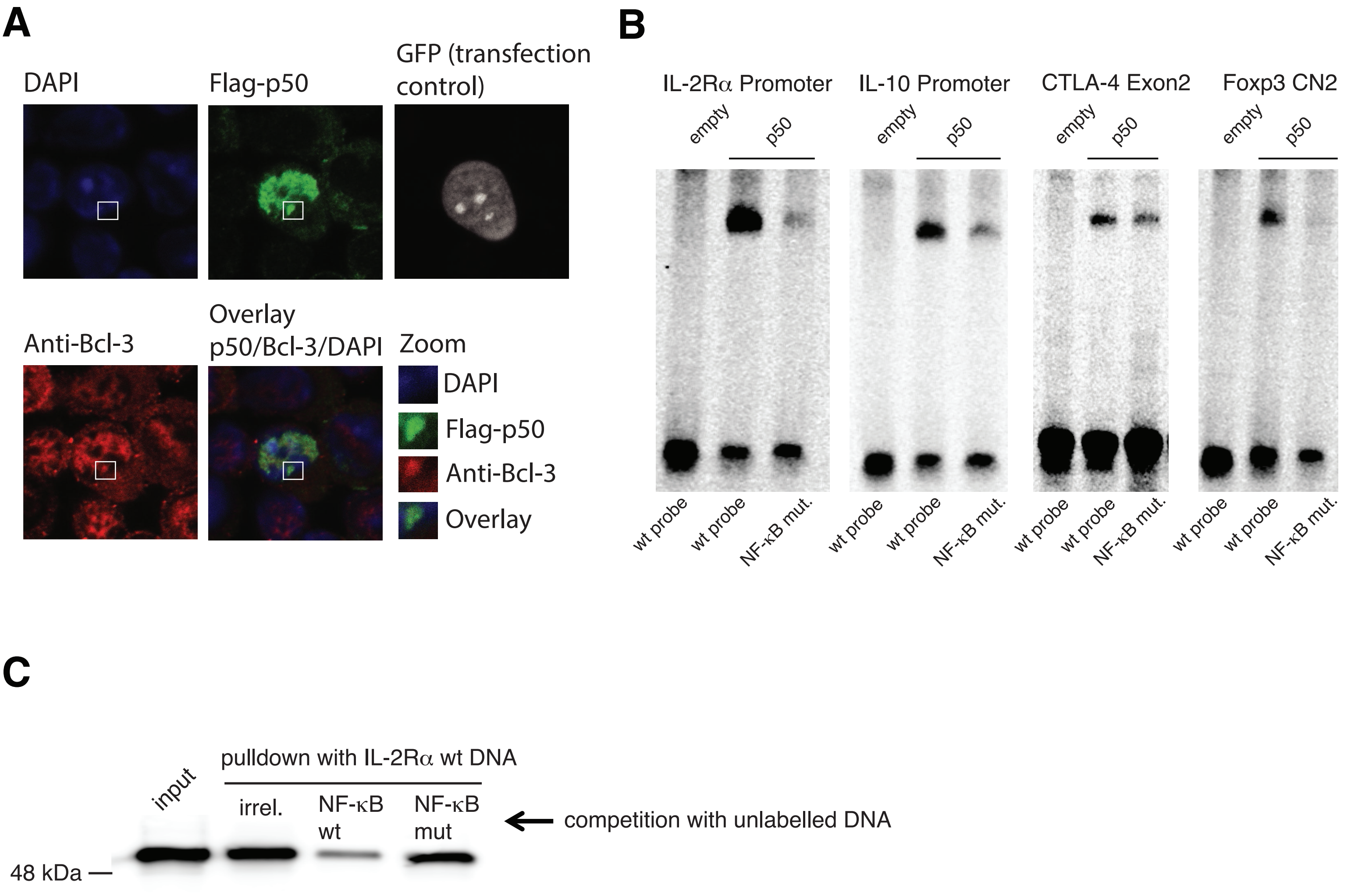

Supplementary Figure 6: Bcl-3 inhibits binding of p50/p65 to Treg-relevant genes
(a) Confocal microscopy of regulatory T cells from Bcl-3^{TOE} mice with cells electroporated with p50 constructs and GFP as transfection control (gray), restimulated with PMA/Ionomycin, stained for DAPI (blue), Flag-tagged-p50 with anti-Flag-antibodies (green), Bcl-3 with anti-Bcl-3 antibodies (red). Zooms visualize co-localization in the nucleus. (b) Electrophoretic mobility shift assays with nuclear lysates from HEK293T cells expressing p50 to DNA probes from intergenic regions (either promoters or introns that demon strated high conservation and a conserved NF-κB side) of the IL-2 receptor, IL-10, CTLA-4 and Foxp3. Probes were also generated with mutations in the potential NF-κB binding side. One representative of two experiments is shown. (c) Pulldown experiments with IL-2Rα probe using 100x- fold of cold irrelevant DNA or IL-2Rα probe or with mutant probe as indicated.

Supplementary Figure 7

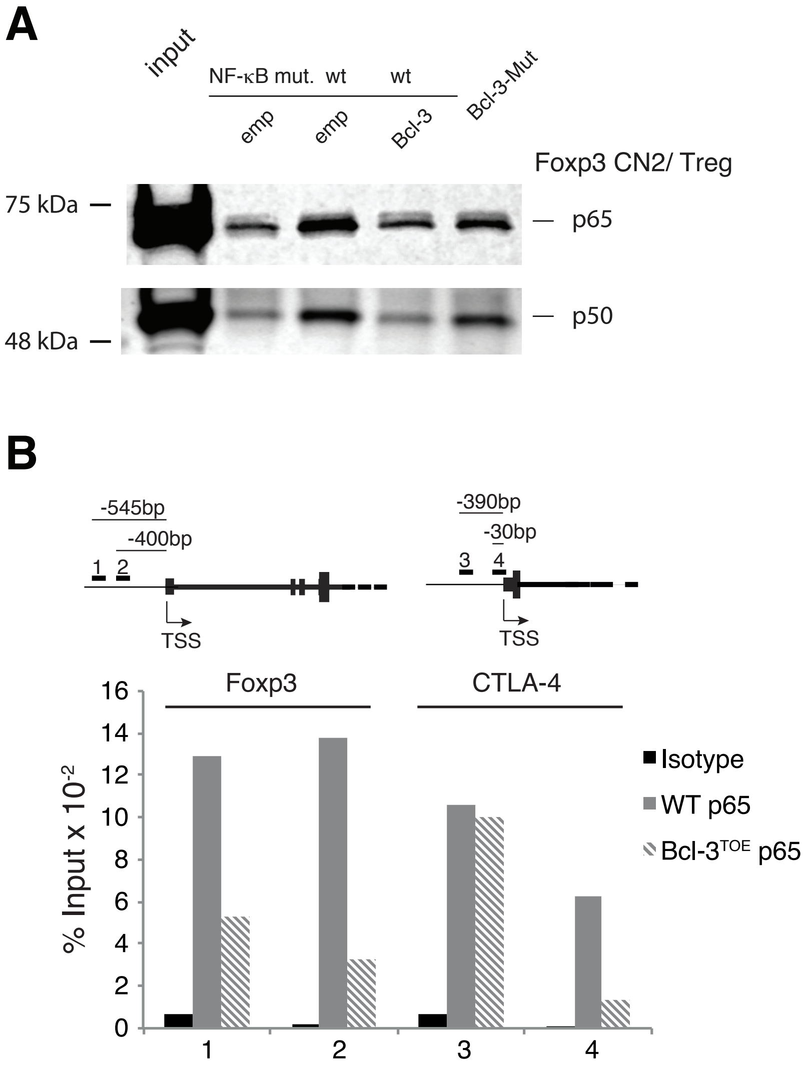

Supplementary Figure 7: The p50/p65 heterodimer is affected in binding to Treg genes by Bcl-3 levels and its interaction to p50

(a) Pull-down experiment with DNA probes of the Foxp3 CN2 region (used in Figure 7D) with protein extracts from Tregs, to which Bcl-3 or empty vector overexpressing lysates were added. One out of two experiments is shown. (b) Chromatin-immunoprecipitations using p65 and isotype control antibodies were performed in in vitro differentiated Tregs from Bcl3^{TOE} mice and littermate controls. Primer were designed as indicated for two sites within the CTLA-4 and Foxp3 Promoter region. One of two representative biological examples is shown.

Supplementary Figure 8

Figure 1 D

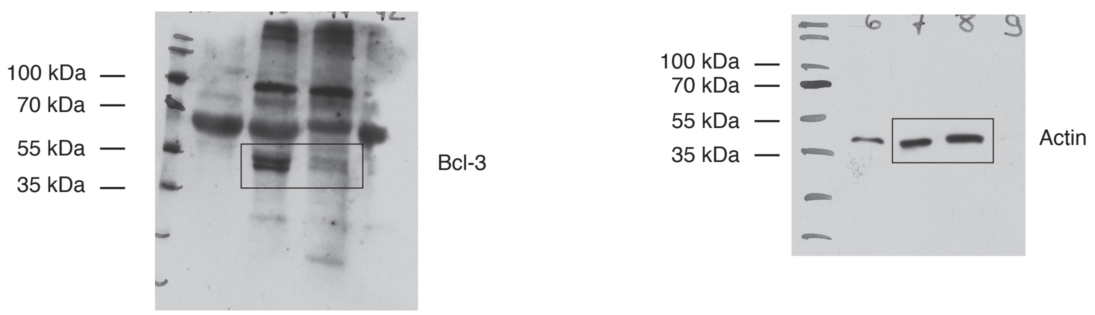

Figure 2 A

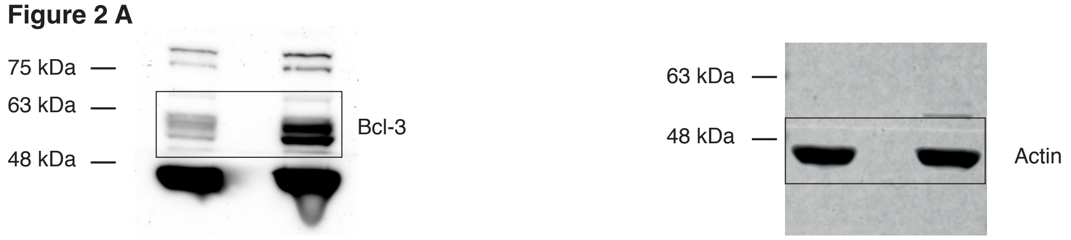

Figure 7 A

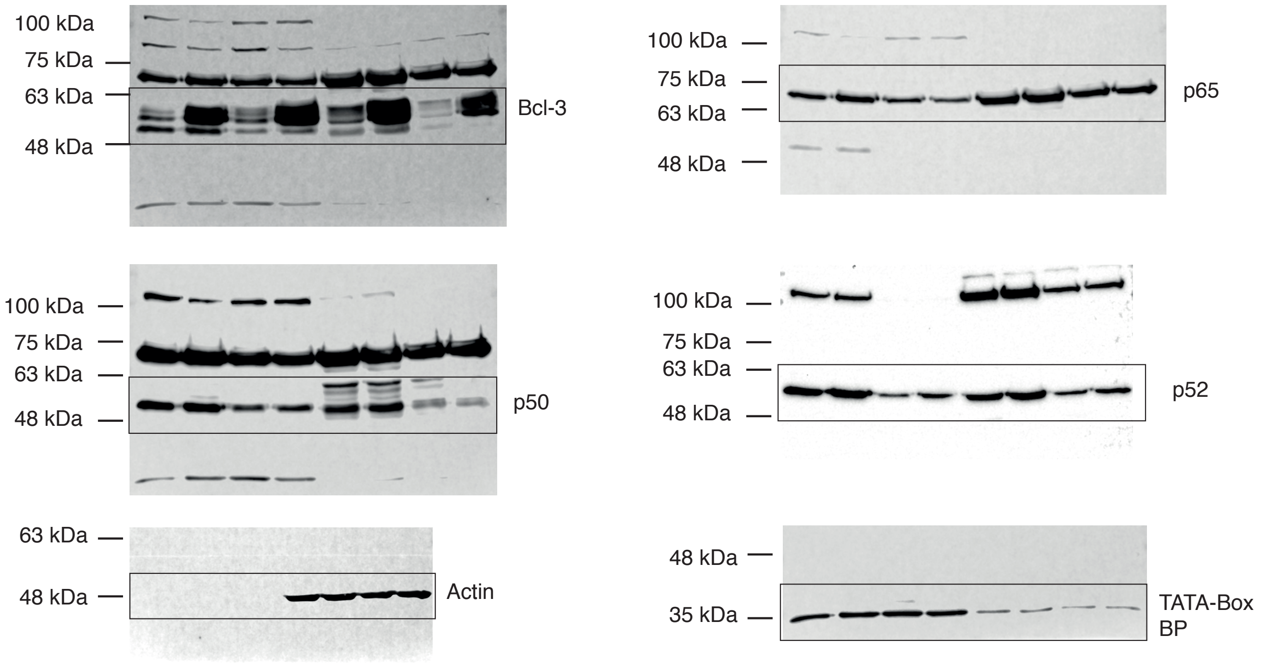

Figure 7 B

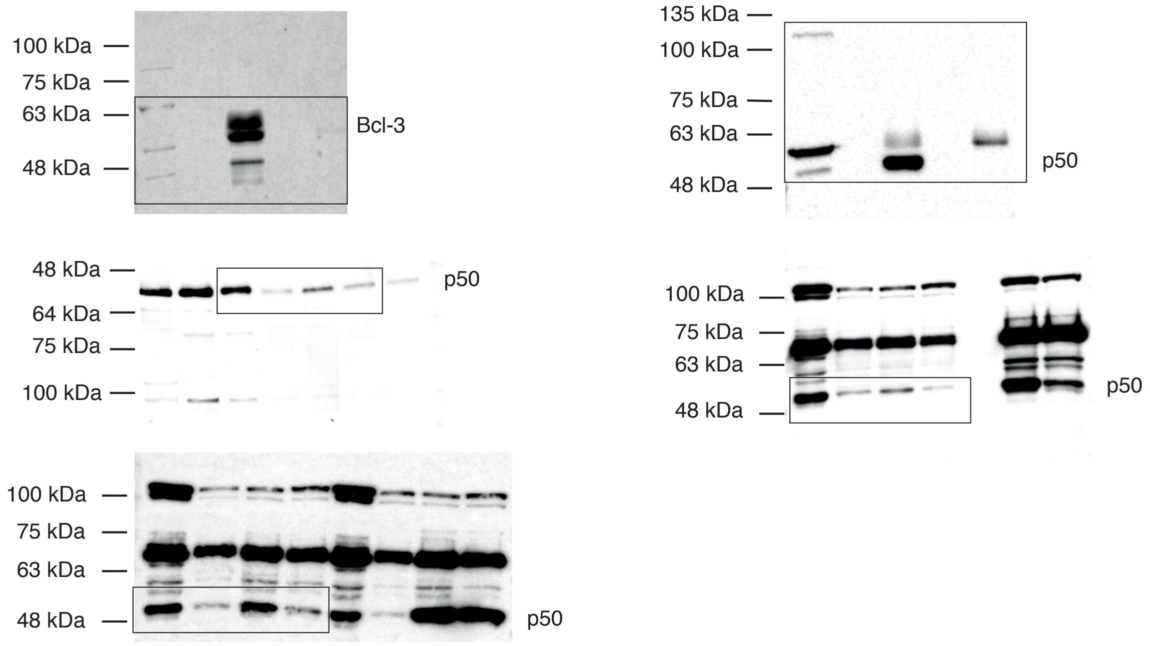

Figure 8 B

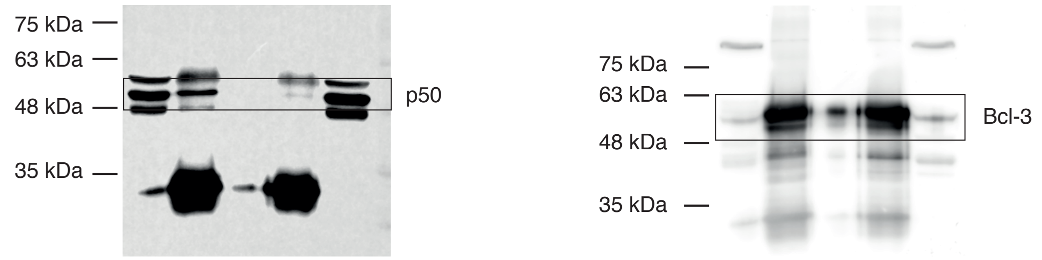

Figure S6 C

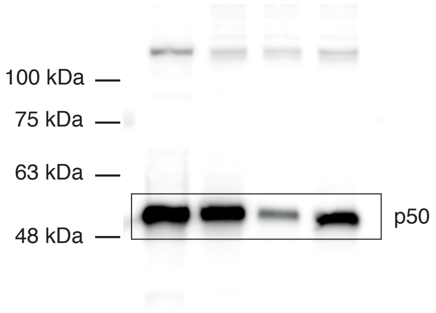

Figure S7 A

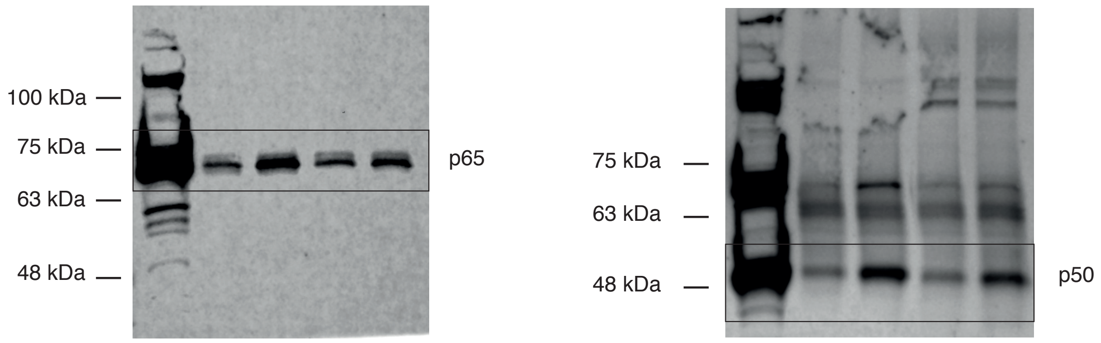

Supplementary Figure 8: Original western blots

The blot areas within the boxes are shown in the figures and supplementary figures, as indicated.

Supplementary Figure 9

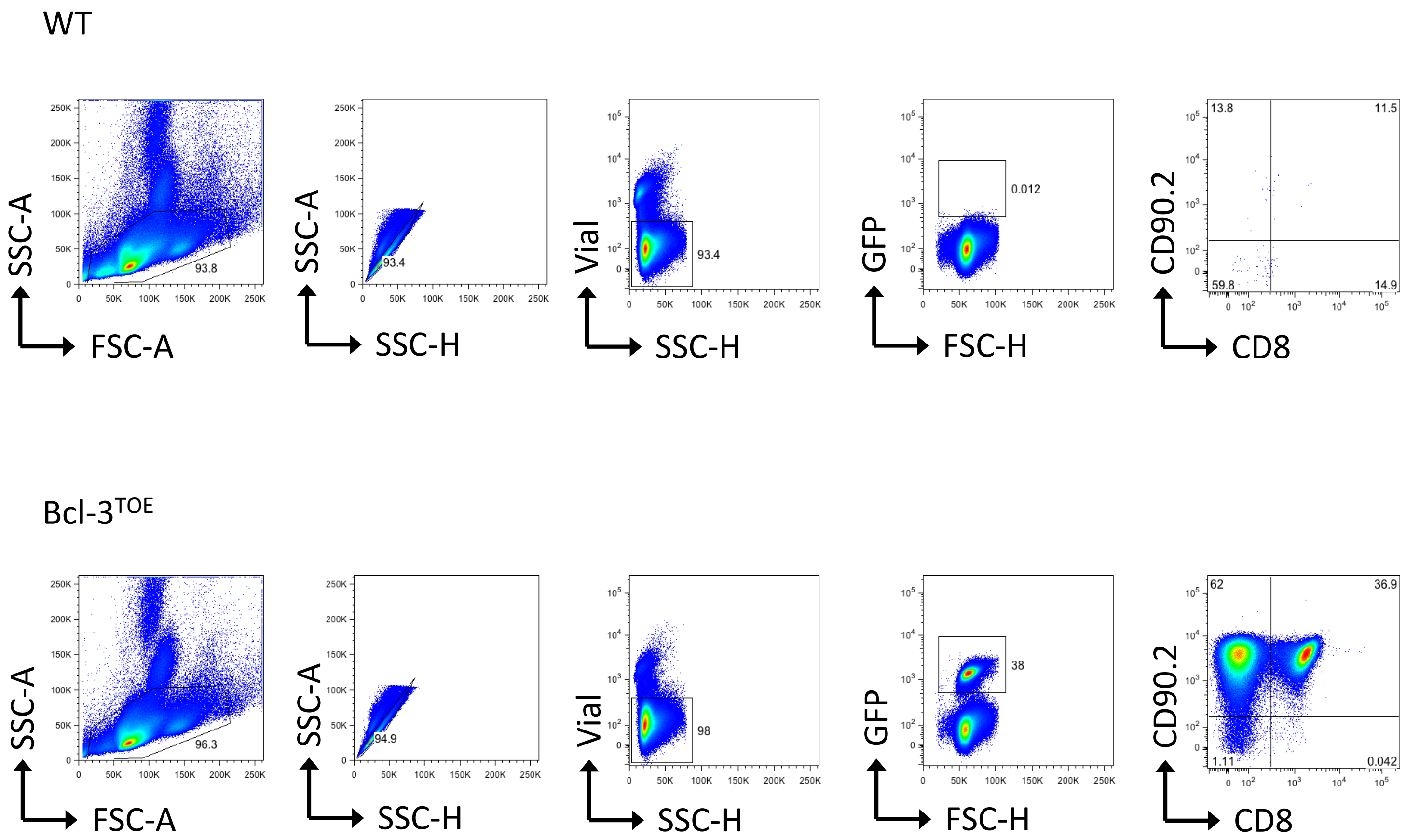

Supplementary Figure 9: Representative FACS gating strategy

Antibody	From	Clone	Dilution	Fluorochrome
CD4	BD	RM4-5	1/200	V500
CD8α	BD	53-6.7	1/200	V450
CD25	BD	PC61	1/200	APC
CD44	eBioscience	IM7	1/1000	PE
CD62L	eBioscience	MEL-14	1/1000	APC
CTLA-4	BD	UC10-4F10-11	1/200	PE
Foxp3	eBioscience	FJK-16s	1/100	APC
GM-CSF	eBioscience	MP1-22E9	1/200	PE
GITR	eBioscience	Dan11mag	1/200	Biotin/PE-Cy7
Helios	Biolegend	22F6	1/100	PE
IL-10	BD	JES5-16E3	1/200	APC
IL-17A	eBioscience	eBio17B7	1/400	V450
IFNg	eBioscience	XMG1.2	1/1000	PE-Cy7
TCRβ	eBioscience	H57-597	1/200	PE
TCR$\gamma\delta$	eBioscience	eBio GL3	1/200	PerCP eFluor 710

Supplementary Figure 10: Table of antibodies utilized
